# New insight into the agonism of protease-activated receptors as an immunotherapeutic strategy

**DOI:** 10.1016/j.jbc.2023.105614

**Published:** 2023-12-29

**Authors:** Yuhong Jiang, Lei Lu

**Affiliations:** 1 Sichuan Engineering Research Center for Biomimetic Synthesis of Natural Drugs, School of Life Science and Engineering, Southwest Jiaotong University, Chengdu, Sichuan, China; 2 School & Hospital of Stomatology, Wenzhou Medical University, Wenzhou, Zhejiang, China

**Keywords:** protease-activated receptors (PARs), agonism, immunotherapy, immune cells, proteases

## Abstract

The activation and mobilization of immune cells play a crucial role in immunotherapy. Existing therapeutic interventions, such as cytokines administration, aim to enhance immune cell activity. However, these approaches usually result in modest effectiveness and toxic side effects, thereby restricting their clinical application. Protease-activated receptors (PARs), a subfamily of G protein-coupled receptors, actively participate in the immune system by directly activating immune cells. The activation of PARs by proteases or synthetic ligands can modulate immune cell behavior, signaling, and responses to treat immune-related diseases, suggesting the significance of PARs agonism in immunotherapy. However, the agonism of PARs in therapeutical applications remains rarely discussed, since it has been traditionally considered that PARs activation facilitates disease progressions. This review aims to comprehensively summarize the activation, rather than inhibition, of PARs in immune-related physiological responses and diseases. Additionally, we will discuss the emerging immunotherapeutic potential of PARs agonism, providing a new strategic direction for PARs-mediated immunotherapy.

Immunotherapy has been emerged as a revolutionary medical treatment that harnesses the body's own immune system to fight cancer and other immune-related diseases, such as infections and autoimmune disorders. However, one of the ongoing challenges for immunotherapy is to enhance its effectiveness and safety for the various types of cancer and diseases, as well as the unique characteristics of individual patients (1). The effectiveness of immunotherapy is heavily dependent on the activity of immune cells; however, activating the immune system to attack harmful cells or pathogens can also cause potential side effects and complications (2, 3, 4). Consequently, understanding the shared activation mechanisms of different immune cells involved in both physiological and pathophysiological processes and the dynamic microenvironments that that favor immune responses is essential for developing novel immunotherapeutic strategies.

The activation and mobilization of immune cells are known to be closely associated with the microenvironment, particularly under pathological conditions, such as presence of pathogens or tissue damage (5, 6, 7). When the body encounters foreign or harmful substances, immune cells become activated and release various immune mediators like cytokines, chemokines, and proteases, etc., that serve to attract other immune cells to the site of infection or injury. Immune cells, including macrophages, neutrophils, natural killer cells, dendritic cells (DCs), and T cells, intricately interact with each other to effectively regulate the intensity and duration of the immune response (1, 8). One of the common approaches for immune activation involves the exogenous administration of cytokines such as interferon (IFN) and interleukins. However, the therapeutic interventions utilizing these cytokines have exhibited only modest effectiveness while inducing considerable toxicity, thereby limiting their clinical application (9, 10).

Proteases play a crucial role in many physiological processes by cleaving specific protein substrates, including blood coagulation, tissue repair, and particularly immune responses during inflammation. Matrix metalloproteinases, for instance, are well known for their role in degrading extracellular matrix components such as collagen and elastin, as well as promoting tissue remodeling and repair. However, they can also activate pro-inflammatory chemokines and cytokines, contributing to the initiation and propagation of inflammation (11). Meanwhile, proteases such as cathepsins generate chemotactic peptides like C5a and CXCL8, attracting immune cells like neutrophils and macrophages to the inflammatory site (11, 12). More importantly, proteases have the ability to communicate with both immune and nonimmune cells directly through a unique set of G protein-coupled receptors (GPCRs) known as protease-activated receptors (PARs) (13). Upon activation, PARs trigger a cascade of intracellular signaling pathways that ultimately lead to changes in gene expression, cytokine production, and immune cell activation. Understanding the activation of PARs presents an opportunity to elucidate the intricate mechanisms that govern immune responses, offering insights for potential therapeutic interventions (Fig. 1).

**Figure 1:**
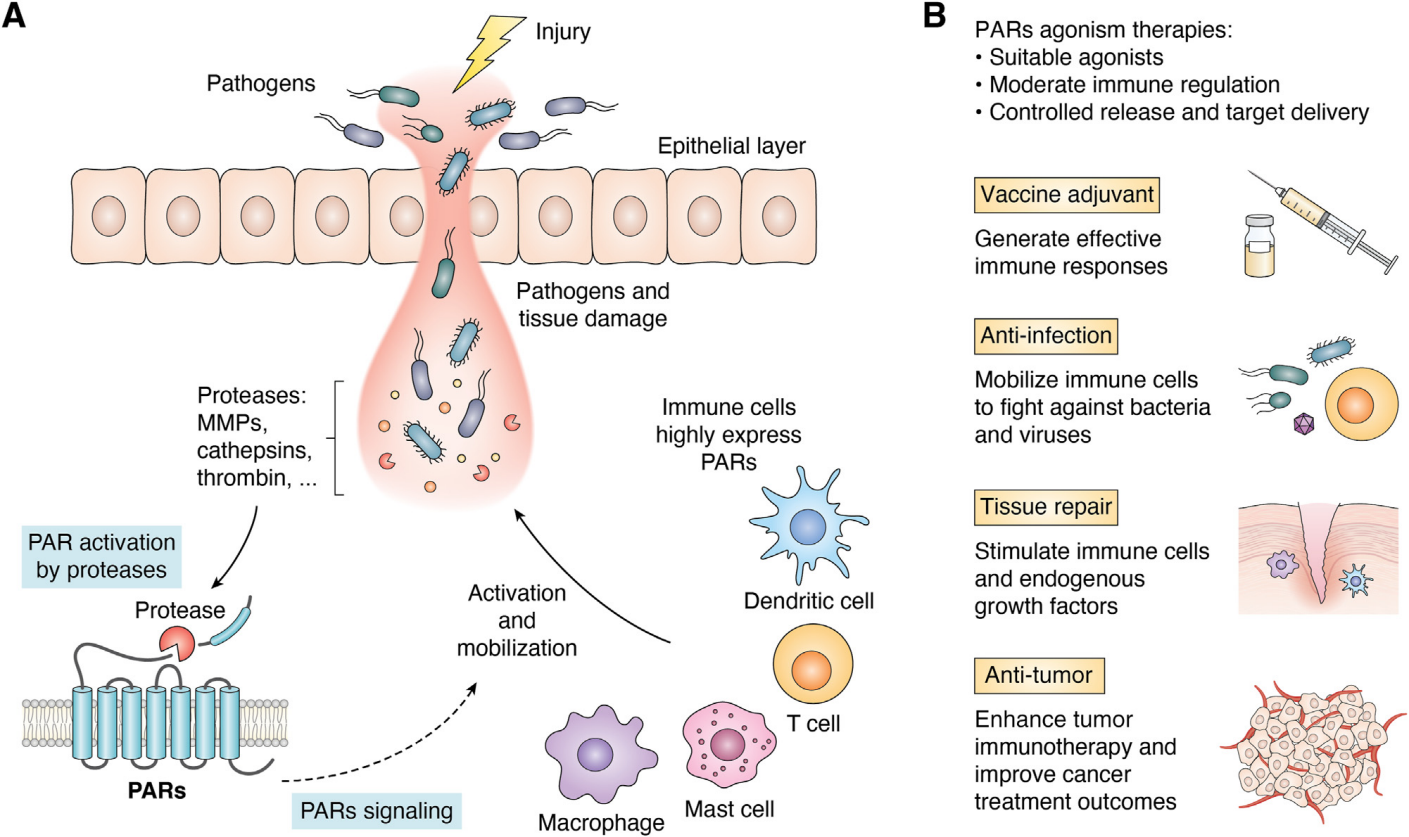
**The utilization of PAR agonism as an immunotherapeutic strategy.** PARs agonism mediated immune modulation upon exogenous stimulation (*A*), and the potential immunotherapeutic strategies inspired by PARs agonism (*B*). MMP, matrix metalloproteinase; PAR, protease-activated receptor.

PARs have been shown to modulated various aspects of immune responses, including inflammation, immunity to infections, and autoimmune diseases. PARs, located on the cell membrane, undergo natural cleavage by proteases like thrombin, trypsin, and tryptase, etc., which allows them to couple to G protein and initiate intracellular signal transduction. Consequently, they modulate immune cell infiltration, DCs maturation, and cytokine release and other immune-related processes (13, 14). PARs activation may be the first step for proteases to mediate physiological responses and diseases, indicating the principal role of PARs in protease-mediated cell functions, particularly immune modulation. Alternatively, synthetic ligands can activate PARs, mimicking the effects of proteases and artificially regulating immune-related signaling and responses (15). Studies have revealed that PARs exhibited positive correlation with bacterial or vital infection, as well as disease progression in immune-related conditions (16, 17). These findings further support the potential of PARs as immunotherapy strategies for treating various diseases, including cancer and virus infection. However, while many reports indicated that PARs can be activated to accelerate various disease progression (14), the focus of therapeutic investigations has predominantly been on PARs blockade rather than agonism. Compared to cytokine-based treatments, activating or boosting the immune system through protease-mediated mechanism was relatively less discussed. The utilization of PARs-mediated activation of immune cells to boost natural immunity against diseases is still in its early stages of development. It is worth noting that targeting PARs seems to possess extensive immune modulating capacity, since it can not only participate in protease-related immune diseases but also modulate various immune cell signaling and functions in response to other stimuli. Thus, by understanding the innate regulatory mechanisms of PARs agonism underlying the immune response within the tissue microenvironment, novel strategies can be developed for more effective immune activation.

## PARs agonism and signaling in immune system

### PARs activation and signaling

PARs as a subfamily of GPCRs are classified into four members, PAR1, PAR2, PAR3, and PAR4 (18). Generally, PARs possess a main structure that consist of seven transmembrane, three extracellular loops, and three intracellular loops. Both endogenous (*e.g.*, thrombin and trypsin) and exogenous (*e.g.*, German cockroach) proteases can specifically cleave the N-terminal domain of PARs at R^36^↓S^37^ site to expose a new N-terminal sequence that acts as a self-ligand, ‘tethered ligand’, which binds to second extracellular loop of the receptor (14). After that, PARs are activated to generate a variety of downstream signal transductions *via* Gα protein or β-arrestin, modulating physiological responses and diseases (19, 20). Classically, PARs coupled to Gα_q_ protein can activate downstream inositol triphosphate or diacylglycerol through phospholipase C-β to stimulate calcium release or PKC, affecting inflammation, immune responses, and nervous system development (20). For Gα_i_/Gα_s_-dependent signaling, Gα_i_ can downregulate cyclic adenosine monophosphate (cAMP) by blocking adenylate cyclase, while Gα_s_ can upregulate cAMP to initiate inflammation and thrombosis (21). Additionally, Gα_12/13_ can regulate roh-member A and ROCK activation, thereby regulating cytoskeleton rearrangement, platelet secretion, and cell hypertrophy (22). Importantly, β-arrestin can couple to PARs in the absence of Gα protein to activate mitogen-activated protein kinase/extracellular signal-regulated kinase (ERK) phosphorylation, promoting tumor growth and immune cell development (23). β-arrestin can also mediate the endocytosis of PARs through PAR-β–arrestin coupling complex or PAR-β–arrestin–G protein coupling complex, initiating endosomal ERK and cAMP signaling (24, 25, 26). Some GPCR kinases can desensitize activated PARs through phosphorylation and β-arrestin binding and promote G proteins to return to the original inactive stages, terminating intracellular signal transductions (27).

Alternatively, some PAR proteases, such as elastase, cathepsin S, can cleave PARs at uncanonical sites to trigger biased activation, which preferably stimulate some specific signaling pathway but do not affect other pathways (28, 29). For instance, elastase activated PAR2 with A^66^↓S^67^ and S^67^↓V^68^ cleavage site to partially initiate ERK1/2 phosphorylation and cAMP accumulation but not calcium release, whereas canonical activation of PAR2 by trypsin can induce all signaling pathways (30). Apart from proteases, synthetic agonists, such as 2f-LIGRL-NH_2_, AY254, have also been developed to activate PARs through mimicking or modifying tethered ligand, which can also stimulate classical or biased signaling (29).

### PARs agonism in immune system

Numerous proteases, especially serine proteases, actively participate in both innate and adaptive immune responses, uncovering their principal roles in immune system (31). As receptors activated by serine proteases, emerging evidence revealed PARs modulation in the protease mediated immune responses and related diseases. Early studies reported that German cockroach proteases activated PAR2 to trigger innate immune response to cockroach allergen through pulmonary neutrophils, cytokine release, or DC recruitment, consequently participating in allergic airway inflammation and related immune cells (32, 33, 34). A recent study has also revealed that Granzyme K induced PAR2 activation to stimulate abundant cytokine releases, including interleukin (IL)-6 and IL-8 (35). Trypsin can also initiate PAR2 activation to mediate IL-17A/IL-17R signaling axis and neutrophilic infiltration when mice were acutely exposed to house dust mite allergens, and the PAR2 antagonist blocked such effects (36). Additionally, it has been reported that mast cell tryptase and PAR2 agonist (SLIGRL-NH_2_) promoted Th1-dependent colonic immune responses and visceral hypersensitivity in postinfectious irritable bowel syndrome (37), while other proteases, such as gelatinase, StmPr1, also activated PAR2-mediated immune activation in inflammatory diseases (38, 39). Cathepsin S also induced PAR2 activation as an immune modulator to trigger immune dysregulation and kidney allograft rejection (40). Caminero *et al.* (41) reported that elastase produced by *Pseudomonas aeruginosa* activated PAR2 to largely affect host immune responses and consequent food sensitivities *via* inducing various cytokine expressions in celiac disease, further indicating the role of PARs in immune systems.

Apart from proteases-induced immune diseases, abundant investigations have uncovered the participation of PARs activation in other immune responses. It has been reported that PAR1 expression positively correlated with expressions of pro-inflammatory cytokines (IL-17A, IL-22, and IL-23A) in colon tissues from patients with Crohn’s disease, suggesting the involvement of PAR1 Th17-related immunity (42). Intriguingly, PAR1-mediated immune regulation can be cell type-dependent, since there were significant difference in Th17-related cytokines, IL-1β and T cell infiltration among a global (PAR1^−/−^), myeloid-(PAR-1^ΔM^), or enterocyte-specific (PAR-1^ΔEPI^) PAR1 deficiency (43). Importantly, a recent study has demonstrated that PAR1 and PAR2 activation restricted the progression of collagen-induced arthritis, whereas PAR1 or PAR2 knock largely deteriorated collagen-induced arthritis *via* regulating immune cell functions and cytokine releases (44). With activation of the coagulation cascade as the host defense response, PARs including PAR1, PAR2, and PAR4 have been also discovered to possess superior protective effects against viral infection like coxsackievirus B3 and influenza virus infection (17, 45, 46). Moreover, there are a variety of receptor transactivations among PARs family or between PARs and another receptor family, cooperatively mediating biological responses and diseases (23, 47, 48, 49). Especially, PARs like PAR1 and PAR2 exhibits extraordinary signal crosstalk with toll-like receptor (TLR)4 (49, 50), while TLR4 is a vital receptor for antigen cross-presentation and immune responses to microbial infection (51, 52). A study has reported that PAR1 or PAR2 exhibited strong interaction with TLRs (TLR2 and TLR4) and nucleotide-binding oligomerization domain receptors in initiating multiple cytokine expressions and innate immune responses to various types of bacteria (53). Therefore, PARs may be collaborated with TLRs to participate in immune system *via* receptor transactivation; however, it still lacks experimental investigations on immune modulation mediated by crosstalk of PARs and TLR. Alternatively, some researchers suggested the potential participation of PARs in severe coronavirus disease 2019, since PARs directly interacted with SARS-CoV-2 and one PAR protease, TMPRSS2, probably involved in mediating coronavirus to enter the host cells (54, 55). PAR activation can also trigger various pro-inflammatory cytokine release, which might be relevant to cytokine storm induced by coronavirus disease 2019, suggesting their immune modulations during coronavirus infection.

## PARs modulate immune cells and immune responses

**Figure 2:**
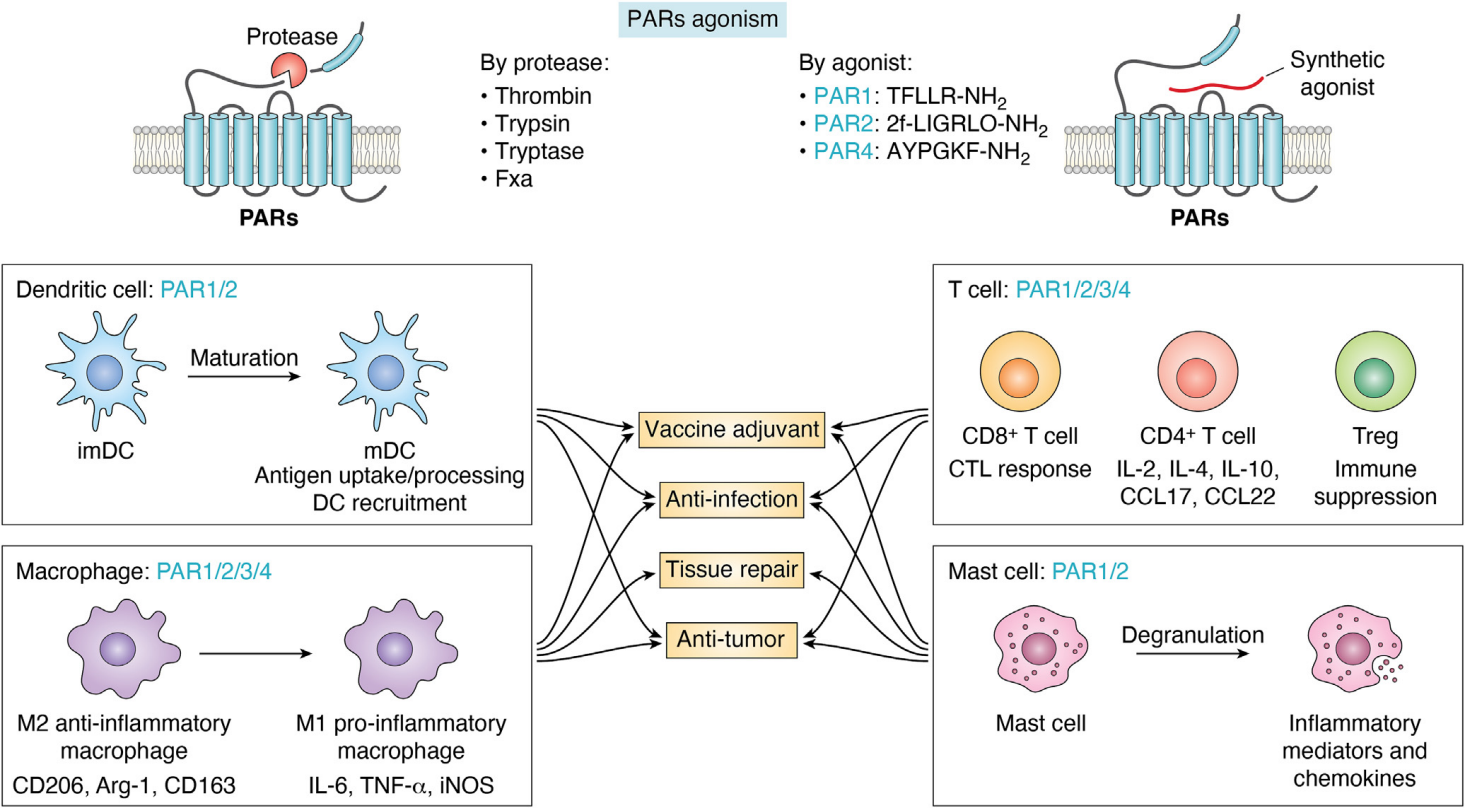
**Different PARs modulate distinct immune responses of DCs, T cells, macrophages and mast cells, triggering different therapeutical applications.** CTL, cytotoxic T lymphocyte; DC, dendritic cell; IL, interleukin; PAR, protease-activated receptor; Treg, regulator T cell.

Immune cells, such as DCs, T cells, macrophages, and mast cells, play principal roles in regulating immune system and preventing infection or disease progression (Fig. 2) (56). PARs as the membrane receptor family are highly expressed on a variety of immune cells and modulate downstream signal transductions and cellular functions (13). Generally, the potency of PARs modulation in immune responses is highly dependent on PAR-mediated immune cell signaling and functions (Table 1).

**Table 1:** PARs agonism-mediated immune cell signaling and functions

PARs	Immune cells	Agonists	*In vivo* study	Signaling and functions	References
PAR1	DCs	Thrombin		Stimulated C3 complement and IL-12/IL-17 production, and Th1 immune responses in kidney transplant recipients *via* PAR1 signaling	(61)
	CD8^+^ T cells		Mice	Accelerated calcium release and modulated CD8^+^ T responses *via* actin polymerization and repositioning of MTOC at the immunological synapse	(69)
	CD8^+^ T cells	Thrombin		Promoted CD8^+^ T cell chemokinesis and cytokine release in HIV-infected patients	(70)
	Th1, Th2, Th17		Mice	Protected from CIA *via* regulating T cells and cytokine release	(44)
		Thrombin	Mice	Exhibited a signal crosstalk with TLR3 to prevent CVB3 level and related myocarditis through p38 activation and IFN-β/CXCL10 expression.	(111)
	Macrophages	TFLLR-NH_2_	Mice	Facilitated poly I:C to induce IFN-β and CXCL10 expression, contributing to the antiviral responses	(45)
		Thrombin, SFLLRN		Initiated Gα_i_/PI3K signaling to inhibit breast cancer cell migration and invasion	(127)
		FXa		Prevented cancer cell migration without the involvement of Gα_i_ protein	(128)
	Monocytes	Thrombin		Induced M2-like macrophages with impaired plasticity towards M1 re-differentiation *via* enhanced IL-4 release.	(74)
	Macrophages	Thrombin		Promoted macrophage M1 polarization *via* PI3K/AKT and NF-κB.	(75)
	Macrophages	Thrombin	Mice	Promoted macrophage M1 polarization *via* IFN-γ.	(76)
	Macrophages	Thrombin	Mice	Induce a unique phenotype of macrophage that appears to resemble the alternatively activated M2a phenotype.	(78)
	Macrophages	Thrombin		Directly induced macrophage recruitment in pancreatic cancer.	(79)
	Macrophages	Plasmin and Thrombin	Mice	Regulates MMP-12 expression.	(81)
	Macrophages	Acanthamoeba proteases	Mice	Increased IL-12 production.	(82)
	Macrophages	TFLLR-NH_2_	Mice	Enhanced the antiviral response in mice *via* the increased expression of IFN-β and CXCL10	(45)
	Macrophages	TFLLR-NH_2_	Mice	Suppressed secretion of IRF5 and IL-12/23 and inhibit both Th1 and Th17 immune responses to *Helicobacter pylori* infection in the gastric mucosa.	(83)
	Mast cells	Thrombin		Increased the expressions of PAR1, PAR2, PAR3, and PAR4 mRNA, as well as the release of VEGF, multiple pro-inflammatory mediators and chemokines.	(95)
	Mast cells	Thrombin		Increased the expression of PAR1 and PAR4 mRNA, as well as pro- and anti-inflammatory mediators.	(96)
	Mast cells	SFLLRN-NH_2_ and TFLLR-NH_2_		Caused degranulation and induced GM-CSF and IL-8 synthesis.	(97)
	Neutrophil		Mice	Regulated CXCL1 expression and neutrophil recruitment, controlling mortality of mice infected with IAV	(112)
			Mice	Promoted tumor growth and metastasis *via* PI3K/AKT/STAT3/NF-κB signaling	(129)
PAR2	DCs	Serine proteases, SLIGRL	Mice	Simulated DC development	(58)
	DCs, CD4^+^ T cells	TFVIIa	Mice	Expressed on DCs mitigated CD4^+^ T-cell priming *via* PAR2 signaling	(59)
	DCs	German cockroach proteases	Mice	Promoted recruitment and/or differentiation of myeloid DCs in the lungs *via* CCL20 and GM-CSF production	(33)
	Th1, Th2, Th17		Mice	Triggered a protective role for restricting CIA *via* regulating T cells and cytokine release	(44)
	DCs, CD4^+^ T cells, CD8^+^ T cells	2f-LIGRLO-NH_2_	Mice	Facilitated DCs transportation to draining lymph nodes, CD4^+^ and CD8^+^ T-cell activation	(60)
	CD4^+^ T cells	Papain	Mice	Promoted antigen presentation to naïve T cells and CD4^+^ Th2 cell differentiation *via* CCL17, CCL22 and IL-4 cytokine release	(67)
	Neutrophils	SLIGRL-NH_2_	Mice	Prevented influenza virus type A *via* regulating IFN-γ and RANTES production	(46)
	CD8^+^ T cells, Memory T cells	SLIGRL-NH_2_	Mice	Promoted CD8^+^ T cells and T effector memory cells to protect mice from lethal influenza virus	(107)
			Mice	Decreased TLR3-dependent IFN-β expression activation to modulate innate immune response to CVB3 infection	(114)
	Macrophages		Mice	Modulated IL6, IL12p40, TNF-α or CXCL-2 productions against HBV or IAV	(115, 116)
	MDSCs, TAM, CD4^+^ T cells		Mice	Inhibited MDSCs and TAM, and promoted antitumor CD4^+^ T cells	(108)
	Monocytes	Trypsin		Induces M2-like macrophages with impaired plasticity towards M1 re-differentiation *via* enhanced IL-4 release.	(74)
	Macrophages	2f-LIGRLO-NH_2_		Induces M1 macrophage polarization through the FOXO1-dependent pathway.	(77)
	Macrophages	FXa		Triggers pro-inflammatory responses in RAW 264.7 macrophages *via* the ERK1/2 pathway.	(80)
	Macrophages		Mice	Increased the efficient phagocytosis of *Pseudomonas aeruginosa via* cAMP signaling	(84)
	Macrophages	SLIGRL-NH_2_	Mice	Enhanced the LPS-induced phagocytic activity and NO, ROS, and cytokine production.	(85)
	Mast cells	SLIGKV-NH_2_ and 2f-LIGRLO-NH_2_		Caused degranulation and induced GM-CSF and IL-8 synthesis.	(97)
		*Candida albicans* Sap6		Regulated p38 MAPK signaling and IL-8 production, playing a role in *C. albicans* oral infection	(113)
PAR3	Treg	Protease-activated protein C	Mice	Restricted T cell activation to prevent graft-*versus*-host disease *via* PAR2 and PAR3 heterodimer	(68)
PAR4	Treg		Mice	Impaired function, stability, and mobility of Tregs by excessive PAR4 signaling. PAR4 stimulated PI3K/AKT signaling to modulate Foxp3, CD25, and CD62L *via* upregulation of mTOR and downregulation of Foxo1 and PTEN.	(71)
	Treg		Mice	Modulated posttraumatic activation of CD4^+^ FoxP3^+^ Tregs	(72)
	Macrophage, NK cells	Thrombin, AYPGKF	Mice	Regulated TLR3-dependent IFN-β, CXCL10, CCL5, CXCL1 and MCP1 to protect mice from CVB3 and H1N1 IAV infection	(17)
	Macrophages	AYPGKF-NH_2_	Mice	Enhanced the LPS-induced phagocytic activity and NO, ROS, and cytokine production.	(86)
			Mice	Suppressed tumor growth and metastasis *via* NF-κB signaling	(129)

Abbreviations: cAMP, cyclic adenosine monophosphate; CCL20, chemokine ligand 20; CIA, collagen-induced arthritis; CVB3, coxsackievirus B3; ERK, extracellular signal-regulated kinase; FXa, Factor Xa; GM-CSF, granulocyte-macrophage colony stimulating factor; HBV, hepatitis B virus; IAV, influenza A virus; IL, interleukin; LPS, lipopolysaccharide; MAPK, mitogen-activated protein kinase; MDSCs, myeloid-derived suppressor cells; MMPs, matrix metalloproteinases; MTOC, microtubule organizing center; NK, natural killer cells; NO, nitric oxide; PI3K, phosphoinositide 3 kinase; ROS, reactive oxygen species; TAM, tumor-associated macrophages; TFVIIa, tissue factor VIIa; TLR, toll-like receptor; VEGF, vascular endothelial growth factor.

### DCs

DCs as professional antigen-presenting cells are the vital modulator for innate and adaptive immune responses (6). DCs are in charge of antigen uptake and processing and present antigens to T cells for immune activation (57). Serine proteases can be danger stimulus for initiating DC maturation with enhanced expressions of major histocompatibility complex molecules and costimulatory signals to T cells. Among PARs family, PAR2 has been uncovered to participate in regulating DC maturation and functions (33, 58, 59, 60), for instance, PAR2 has been identified to be a principal receptor to mediate protease-induced DC development in immune system (58). Tissue factor VIIa expressed on DCs modulated CD4^+^ T-cell proliferation and cytokine release *via* PAR2 signaling (59), while German cockroach proteases activated PAR2 to stimulate chemokine ligand 20 and granulocyte-macrophage colony stimulating factor production in the airways and to facilitate DC recruitment to the lungs for initiation of allergic airway responses (33). The synthetic agonist, 2f-LIGRLO-NH_2_, can also induce PAR2 activation to transport DCs to draining lymph nodes and remarkably enhanced CD4^+^ and CD8^+^ T-cell activation in immune system (60). Alternatively, thrombin can activate PAR1 expressed by graft-infiltrating myeloid DCs, notably increasing C3 complement production, IL-12/IL-17 cytokine expressions, and Th1 immune responses in kidney transplant recipients (61). Alternatively, it has recently been reported that the cysteine protease papain initiated transient receptor potential vanilloid subfamily 1-dependent CD301b^+^ DC migration and Th2 cell differentiation to modulate allergic immune responses (62), while papain is a classical protease for PARs (PAR2, PAR4), and PARs can sensitize transient receptor potential vanilloid subfamily 1 channel (63), suggesting the role of PARs in papain-mediated DC functions. TLR2 also promoted DCs to initiate antigen cross-presentation to CD8^+^ T cells and consequently triggered cytotoxic T lymphocyte responses, which is highly related to MyD88/JNK/ERK signaling (64). Unsurprisingly, TLR family can also induce transactivation with PARs, and ERK signaling is the main signaling pathways for PARs (29, 49, 50), further indicating the potential participation of PARs in regulation of DCs *via* receptor crosstalk. PARs agonism can stimulate DC activation and maturation to present antigens to T cells, consequently initiating antigen-specific immune responses.

### T lymphocytes

T lymphocytes, such as CD4^+^ T cells, CD8^+^ T cells, and regulator T cells (Tregs), with robust effector functions are essential for initiating immune activation and maintaining immune toleration (65, 66). Cysteine protease allergen papain can promote basophils to present antigen to naïve T cells and CD4^+^ Th2 cell differentiation *via* CCL17, CCL22, and IL-4 cytokine production by PAR2 activation (67), whereas PAR2 activation on DCs could also prevent CD4^+^ T cell proliferation and activation (59), suggesting the involvement of PAR2 in modulating CD4^+^ T cells. PARs crosstalk also contributed to modulation of T cells, since protease-activated protein C can ameliorate T cell reactivity *via* PAR2 and PAR3 heterodimer on Tregs to mitigate the progression of graft-*versus*-host disease (68). Additionally, PAR1 preferably participates in regulation of CD8^+^ T cell activation, differentiation, and functions. It has been reported that activation of PAR1 expressed on CD8^+^ T cells could modulate cytotoxic T lymphocyte responses *via* repositioning of the microtubule organizing center at the immunological synapse (69). Moreover, PAR1 expression was largely upregulated on CD8^+^ T cells from HIV-infected patients and thrombin could activate PAR1 to accelerate chemokinesis of CD8^+^ T cells and pro-inflammatory cytokine release in adaptive immunity (70). Interestingly, a recent study probed the new role of PAR4 in modulation of Treg functions (71). Unlike CD4^+^ and CD8^+^ T cells, Tregs as immunosuppressive cells are representative for immune toleration and homeostasis (66). PAR4 activation initiated the enhancement of mTOR and downregulation of Foxo1 and PTEN *via* phosphoinositide 3 kinase (PI3K)/AKT signaling, controlling the expression of Foxp3, CD25, and CD62L. When abundant proteases accumulated at the inflammatory sites, PAR4 signaling on Tregs turned to be excessive, consequently impairing function of Tregs (71). PAR4 signaling on platelets also regulated posttraumatic activation of CD4^+^ FoxP3^+^ Tregs, indicating its essential role in the interaction between platelets and CD4^+^ Tregs (72). Therefore, PARs actively participate in regulating a variety of T cells with different manners to evoke antigen-specific immune responses, and targeting PARs can be a potential strategy for T cell-related immunotherapy.

### Macrophages

Macrophages are pivotal cells in the innate immune response which express functionally active PAR1, PAR2, and PAR3, but not PAR4 receptors on their surface (73). Recent evidence indicates that the activation of PAR1 and PAR2 on macrophages could also modulate their phenotypes and functions. For instance, the serine proteases, thrombin and trypsin activate PAR1 and PAR2 receptors in monocytes and induces M2-like macrophages with impaired plasticity toward M1 re-differentiation *via* enhanced IL-4 release (74). Other studies indicate that thrombin-mediated PAR1 signaling promotes macrophage M1 polarization *via* PI3K/AKT and NF-κB (75), as well as IFN-γ (76). It also been demonstrated that activation of PAR2 induces M1 macrophage polarization through the FOXO1-dependent pathway (77). Recent study has revealed that the unique phenotype of macrophage stimulated by thrombin appears to resemble the alternatively activated M2a phenotype (78). Specifically, PAR1 signaling directly induced macrophage recruitment in pancreatic cancer (79). Coagulation protease factor Xa triggers pro-inflammatory responses in RAW 264.7 macrophages through the activation of PAR2 *via* the ERK1/2 pathway (80). Matrix metalloproteinase-12 expression from macrophage is regulated by plasmin and thrombin-mediated PAR1 activation (81). Furthermore, PARs also deeply involve in macrophage-mediated innate immunity against various infections. For example, Acanthamoeba trophozoite proteases contribute to the activation and IL-12 production of macrophages through PAR1, but not PAR2 (82). The activation of PAR1 enhanced the antiviral response in mice *via* the increased expression of IFN-β and CXCL10 (45). During chronic gastritis, PAR1 activation suppressed secretion of IRF5 and IL-12/23 by macrophages by which inhibit both Th1 and Th17 immune responses to *Helicobacter pylori* infection in the gastric mucosa (83). The PAR2-mediated cAMP-Rac1 pathway has been shown to play a crucial role in triggering the phagocytosis of *P. aeruginosa* by alveolar macrophages (84). The PAR2 agonist peptide (SLIGRL-NH_2_) (85), as well as PAR4 agonist peptide (AYPGKF-NH_2_) (86), enhanced the lipopolysaccharide-induced inflammatory mechanisms, including the phagocytic activity and nitric oxide, reactive oxygen species, and cytokine production in macrophages. Therefore, PARs may represent a potential target for macrophage-related therapies, such as anticancer and anti-infection, improve the efficacy of vaccines and promote wound healing, by manipulating the polarization and differentiation of macrophages by PARs agonism.

### Mast cells

Mast cells are mainly known for their involvement in allergic reactions by releasing pro-inflammatory mediators and cytokines and proteases. These cells also play crucial roles in many biological processes, including immune regulation, pathogen clearance, and angiogenesis, thereby it has raised attention to harness their therapeutical potential through controlled and localized activation (87, 88). Specifically, mast cells play a vital role in immune modulation by releasing chemical mediators that affect blood vessels, recruit immune cells, and influence the activation and differentiation of other immune cells, including T cells, B cells, and DCs, etc. (89, 90). For PARs agonism, mast cell is best known for its protease tryptase as a potent stimulant for PAR2 on various cell types (91, 92, 93, 94). However, activation of PARs in mast cells can modulate their activity in various ways, such as promoting cytokines release, cell migration, and degranulation. The expressions of PAR1, PAR2, PAR3, and PAR4 mRNA, as well as the release of vascular endothelial growth factor, multiple pro-inflammatory mediators, and chemokines were increased by thrombin stimulation in P815 mouse mast cells, partially though PAR1 (95). Similar result was shown in human mast cell HMC-1, the expression of PAR1 and PAR4 mRNA as well as pro- and anti-inflammatory mediators were increased after thrombin incubation (96). It also been indicated that human skin-derived mast cells can be activated by both PAR1 and PAR2. The PAR1-activating peptides (SFLLRN-NH_2_ and TFLLR-NH_2_) and PAR2-activating peptides (SLIGKV-NH_2_ and 2-furoyl-LIGRLO-NH_2_) cause degranulation and induce granulocyte-macrophage colony stimulating factor and IL-8 synthesis in human skin-derived mast cells (97). Future studies should verify therapeutic potential of PAR agonists for vaccine adjuvant development (98), defense against solid tumors (99, 100, 101, 102), enhance tissue repair (88, 103), and anti-infections (104) in which mast cells are involved.

## The immunotherapeutic effects of PARs agonism

PARs have been identified as potential targets for immunotherapy because of their roles in modulating the immune response. So far, the vast majority research of PARs-mediated immune responses focused on PARs inhibition by antagonists, thereby reducing inflammation and preventing tissue damages, for instance, GB88 and AZ3451 as PAR2 antagonists alleviated PAR2-mediated inflammatory responses and paw edema (105, 106). However, currently, PARs agonism gradually showed the potential to offer effective treatments by mobilizing immune cells for some diseases, especially for viral infections and cancer therapies (45, 46, 107, 108).

### PARs agonism in pathogen infection immunotherapy

Immune responses confer the first line of host defense against a variety of viral or bacterial infections, while the activation of coagulation system is also protective response to restrict pathogen infection (109, 110). As a coagulation protease, thrombin is the vital protease for activating PARs signal transduction and related functions, indicating the role of PARs in pathogen infection. Intriguingly, numerous investigations have reported that PARs activation can effectively trigger protection from a variety of viral infections (17, 45, 46), revealing their therapeutical potentials. Thrombin-activated PAR1, which exerted a signal crosstalk with TLR3, to decrease level of coxsackievirus B3 and related myocarditis through p38 activation and cytokine release (111), while PAR1 activation notably improved expressions of IFN-β and CXCL10 to facilitate poly I:C-initiated antiviral responses (45). The global deficiency of PAR1 directly led to the enhancement of CXCL1 expression and neutrophil recruitment, consequently improving the mortality of mice infected with influenza A virus, supporting the positive participation of PAR1 in immune responses for restricting viral infection (112). PAR4 also modulated TLR3-dependent cytokines to prevent coxsackievirus B3 and H1N1 influenza A virus infection (17). SLIGRL-NH_2_ as a PAR2 agonist powerfully upregulated IFN-γ production and downregulated neutrophils and RANTES release to trigger strong prevention against influenza virus (46). More importantly, this PAR2 agonist peptide can be an excellent mucosal adjuvant for protecting mice from lethal influenza virus *via* initiating CD8^+^ lung T cells and T effector memory cells (107). It is not hard to uncover that PARs agonism can be superior therapeutical strategy for treating viral infection and related diseases. Alternatively, PARs played an essential role in preventing bacterial infection; for instance, PAR2 activation accelerated the *P. aeruginosa* clearance *via* regulating cAMP signaling cascade in innate immunity (84). Moreover, *Candida albicans* hyphae enhanced the expression of PAR1, PAR2, and PAR3, while *C. albicans* Sap6 could activate PAR2 to trigger p38 mitogen-activated protein kinase signaling and IL-8 release, indicating PAR2 as a potential molecular target for preventing *C. albicans* oral infection (113). In contrast, it has been reported that PAR2 activation alleviated innate immune response to coxsackievirus B3 infection *via* TLR3-dependent IFN-β expression (114) and also promoted productions of pro-inflammatory cytokine against hepatitis B virus and influenza A virus (115, 116). Another study also showed that PAR1 activation was beneficial for respiratory syncytial virus, while PAR1 inhibition triggered protective capacity with decreased viral replication (117). It suggested that PARs might exert two opposite effects for immune regulation in different situations of viral infection, which is similar with their Yin-Yang roles in inflammatory responses (19). Therefore, the therapeutic strategy of PAR agonism should be precisely designed and applied for preventing viral infection, according to the certain environment.

### PARs agonism in cancer immunotherapy

Cancer immunotherapy that activates immune systems to fight with cancer becomes one of the most attractive anticancer therapeutical strategies in recent decades. Unsurprisingly, plenty of immune cells in microenvironment around tumor cells actively participate in modulating tumor progression *via* triggering or suppressing immune responses. While immune cells traditionally initiate antitumor immunity, some immunosuppressive immune cells, such as Tregs and tumor-associated macrophages, can remarkedly assist tumor cells to escape from immunological surveillance (118). Especially, tumor cells exert an extraordinary effect for promoting macrophage polarization in tumor microenvironment, leading to the role macrophages transformed from immune activation to suppression (119). PARs overexpressed on a variety of tumor tissues not only involve in regulation of tumor cell functions but also modulate numerous immune cells, including DCs, macrophages, and T cells (120, 121), suggesting the potential roles of PARs in cancer immunity. Importantly, tumor progression is often accompanied by large accumulations of proteases, while proteases, such as thrombin, cathepsin, and matriptase, can initiate degradation of extracellular matrix in tumor microenvironment to facilitate tumor invasion and metastasis (122, 123). Alternatively, proteases, such as kallikrein-related peptidases, exhibited potent antitumor capacities *via* regulating tumor immune microenvironment (124), and these proteases may trigger tumor suppression *via* activating their receptors, PARs. Therefore, targeting PARs can be a therapeutical candidate for cancer immunotherapy.

While there are several investigations have uncovered PARs as a protumor molecular target (125, 126), their modulations in immune cells also indicate that PARs-mediated immune responses in tumor environment may contribute to prevention of tumor progression, which may be a new research direction for both PARs and cancer immunotherapy. PAR2 agonism has been reported to trigger antitumor immunity in colitis-associated tumorigenesis. PAR2 knockout mice exhibited robust enhancement of immunosuppressive cells, including myeloid-derived suppressor cells and tumor-associated macrophage and largely alleviated antitumor CD4^+^ T cell functions in tumor microenvironment, leading to promotion of tumor progression (108). Thrombin or SFLLRN as a peptide agonist can activate PAR1, but not PAR2 or PAR4, to mitigate breast cancer cell migration and invasion *via* Gα_i_/PI3K-depending signaling (127), while coagulation factor Xa attenuated cancer cell migration *via* PAR1 without the involvement of Gα_i_ protein (128). The agonism of PARs may modulate immune cell functions and induce immunosuppressive cells converted to activate immune responses to tumor. Interestingly, a recent study has reported that PAR1 exhibited superior protumor capacity *via* regulating PI3K/AKT/STAT3/NF-κB signaling whereas PAR4-mediated NF-κB activation notably alleviated tumor growth and metastasis, suggesting the opposite effects of PAR1 and PAR4 in tumor modulation (129). Unsurprisingly, multiple and complicated functions of proteases and immune cells in tumor microenvironment contribute to distinct roles of PARs in cancer immunotherapy that are context-dependent.

## Future perspectives

One of the challenges in developing effective immunotherapies based on PARs agonism is identifying and/or expanding the appropriate application scenarios. Immunotherapy enhanced by directly stimulating PARs has emerged as a promising approach to treating various diseases, through activating the immune system to target cancer cells and other foreign invaders. Although there have been few reports, PAR agonists have shown great potential in treatments of viral infection (46, 107) and cancer. Moreover, the activation of PARs robustly stimulates various immune cell mobilization, such as immune cell infiltration, DC maturation, macrophage polarization, and T cell recruitment (Table 1). This suggests that the therapeutic potential of utilizing PARs agonism is not limited to the infection and cancer therapies. The following areas may be future research directions for utilizing PARs agonism in immunotherapies: (i) PARs agonists may act as immune adjuvants to assist antigens to exert antigen-specific immune responses in a variety of vaccines due to their excellent immunoenhancing effects. Probing the adjuvant mechanism of distinct PARs in different vaccine types could assist PAR agonists to be effectively applied in immunotherapy; (ii) PARs activation may promote tissue repair and regeneration by stimulating immune cells and endogenous growth factors. While the immune-related mechanism is not fully understood, some studies reported that PARs play a role in mediating immune cell functions that contribute to tissue wound healing (130, 131); (iii) PARs agonism could be a potent strategy against bacterial infections, particularly those caused by antibiotic-resistant bacteria. By mobilizing immune cells through PAR activation, it may be possible to stimulate the immune system to fight off infections without reliance on antibiotics (84, 85, 132); (iv) PARs agonism have shown powerful protective effects against virus, but they have also been discovered to facilitate viral infections in some cases, suggesting PARs-mediated antiviral response is context-dependent. Probing this controversy is beneficial for valid usage of PARs agonism in antiviral therapeutics; (v) PARs agonism may enhance tumor immunotherapy *via* stimulating immune cell mobilization in tumor immune microenvironment. However, there is still a contradiction between protumor and antitumor effects of PARs agonism, which was context-dependent. Obviously, it needs deep exploration for revealing the distinct roles of PARs activation in specific situations.

To advance PARs agonism therapy toward clinical application, several issues need to be addressed: (i) Developing suitable agonists. While proteases are natural stimuli for PARs, excessive protease activity can be harmful to the immune system. The excessive protease activity can lead to tissue damage and chronic inflammation, which can impair immune function over time (133, 134). Thus, the development of small molecular PARs agonists as therapeutic agents to initiate immune cell activation is necessary. Currently, although numerous synthetic agonists, such as 2f-LIGRLO-NH_2_, AY254, have been developed to exert potent agonism capacity, there is a lack of systemic investigations on their pharmacokinetics and bioavailability *in vivo*; (ii) moderate immune regulation. While PARs agonism can initiate robust immune responses for disease treatment, maintaining immune homeostasis cannot be ignorant. Excessive immune responses triggered by PARs activation may lead to intense immune-related side effects and uncontrollable inflammation, leading to the failure of immunotherapy; (iii) controlled release and targeted delivery. To enhance drug efficiency and minimize adverse effects, PARs agonists can be modified for precise delivery or controlled release. Encapsulating PAR agonists in nanoparticle systems, for example, can target specific immune cells or tissues, reducing systemic toxicity, and improving immunotherapeutic effects. In summary, PARs agonism shows potential for immunotherapy in various disease settings. Understanding the appropriate application scenarios and addressing issues such as agonist development, immune regulation, and targeted delivery will be crucial for advancing this approach toward clinical use.

## Conflict of interest

The authors declare no conflicts of interest with the contents of this article.
